# The effect of gesture expressivity on emotional resonance in storytelling interaction

**DOI:** 10.3389/fpsyg.2024.1477263

**Published:** 2024-12-27

**Authors:** Christoph Rühlemann, James Trujillo

**Affiliations:** ^1^Deutsches Seminar - Germanistische Linguistik, University of Freiburg, Freiburg, Germany; ^2^Institute for Logic, Language and Computation, University of Amsterdam, Amsterdam, Netherlands

**Keywords:** gesture kinematics, emotional resonance, talk-in-interaction, storytelling, electrodermal activity

## Abstract

The key function of storytelling is a meeting of hearts: a resonance in the recipient(s) of the story narrator’s emotion toward the story events. This paper focuses on the role of gestures in engendering emotional resonance in conversational storytelling. The paper asks three questions: Does story narrators’ gesture expressivity increase from story onset to climax offset (RQ #1)? Does gesture expressivity predict specific EDA responses in story participants (RQ #2)? How important is the contribution of gesture expressivity to emotional resonance compared to the contribution of other predictors of resonance (RQ #3)? 53 conversational stories were annotated for a large number of variables including Protagonist, Recency, Group composition, Group size, Sentiment, and co-occurrence with quotation. The gestures in the stories were coded for gesture phases and gesture kinematics including Size, Force, Character view-point, Silence during gesture, Presence of hold phase, Co-articulation with other bodily organs, and Nucleus duration. The Gesture Expressivity Index (GEI) provides an average of these parameters. Resonating gestures were identified, i.e., gestures exhibiting concurrent specific EDA responses by two or more participants. The first statistical model, which addresses RQ #1, suggested that story narrators’ gestures become more expressive from story onset to climax offset. The model constructed to adress RQ #2 suggested that increased gesture expressivity increases the probability of specific EDA responses. To address RQ #3 a Random Forest for emotional resonance as outcome variable and the seven GEI parameters as well as six more variables as predictors was constructed. All predictors were found to impact Eemotional resonance. Analysis of variable importance showed Group composition to be the most impactful predictor. Inspection of ICE plots clearly indicated combined effects of individual GEI parameters and other factors, including Group size and Group composition. This study shows that more expressive gestures are more likely to elicit physiological resonance between individuals, suggesting an important role for gestures in connecting people during conversational storytelling. Methodologically, this study opens up new avenues of multimodal corpus linguistic research by examining the interplay of emotion-related measurements and gesture at micro-analytic kinematic levels and using advanced machine-learning methods to deal with the inherent collinearity of multimodal variables.

## 1 Introduction

Arguably the most fundamental distinction between us is that we all have our own body. Despite this divide—or because of it—we seek to pull others closer to us or be pulled closer to them as a way to facilitate a socioemotional connection with one another (Marsh et al., 2009, p. 334).

Closing the gap between bodies can be achieved in innumerable ways. In talk-in-interaction people regularly repeat one another’s behavior, often without noticing (Tschacher et al., 2024; Koban et al., 2019): they recycle words others have just used, re-use their grammatical constructions, mimic their co-speech gestures, align with their body postures, adapt their breathing rhythms to their partner’s, etc. This “cross-participant repetition of communicative behavior” (Rasenberg et al., 2020, p. 2) has been referred to under various denominations, including resonance (Tantucci and Wang, 2021), alignment (Fusaroli and Tylén, 2016; Garrod and Pickering, 2009; Rasenberg et al., 2020), interpersonal coordination (e.g., Duran and Fusaroli, 2017; Romero and Paxton, 2023; Schmidt and Richardson, 2008; van Ulzen et al., 2008; Konvalinka et al., 2023), accommodation (Giles et al., 1991), coupling (Goldstein et al., 2015), and synchronization (Koban et al., 2019; Mogan et al., 2017).

Resonance is theorized to be a ubiquitous interpersonal process (Palumbo et al., 2016); its ubiquity likely has its roots in the fundamental functions it serves in interaction. Resonance promotes social bonding (Marsh et al., 2009). It may be cognitively desirable as being in synch with others conserves computational resources by merging self- and other-representations and is thus less costly than being alone (Koban et al., 2019). For example, measuring neural activity during synchronous speech using fMRI, Jasmin et al. (2016) found that “detecting synchrony leads to a change in the perceptual consequences of one’s own actions: they are processed as though they were other-, rather than self-produced.” Resonance may also have a role in establishing common ground (Brone and Zima, 2014), increasing subsequent cooperation and affiliation (Wiltermuth and Heath, 2009), and supporting group cohesion (Jasmin et al., 2016). Even when not explicitly interacting, people tend to passively synchronize with one another at different levels. For example, people in the same room exhibit spontaneous synchrony of non-communicative bodily movements, whether simply sitting in view of one another (Koul et al., 2023), or sitting and rocking in a rocking chair (Richardson et al., 2007).

But resonance is not only a behavioral phenomenon. Resonance also plays out on the level of Interpersonal autonomic physiology (IAP), defined as “the relationship between people’s physiological dynamics, as indexed by continuous measures of the autonomic nervous system (ANS)” (Palumbo et al., 2016, p. 99). For example, infants and mothers have been shown to synchronize their respiratory kinematics during phases of increased infant attention as indexed by decelerated heart beat (McFarland et al., 2020). Mothers’ facial skin temperature was found to align with their infant’s temperature when they watched their child participate in a series of play and stress phases through a one-way mirror (Ebisch et al., 2012). In married partners, respiratory sinus arrhythmia (RSA), the heart rate variation that occurs during the breathing cycle, was found to positively correlate with self-reported marital conflict (Gates et al., 2015). Passive listeners in public performances of classical music exhibited synchrony on a number of physiological measures (Tschacher et al., 2024), including Electrodermal Activity (EDA), a measure that is particularly indicative of affect and emotion.

This capacity of EDA, to index emotional arousal, makes it a valuable tool for research on resonance in *storytelling*. For storytelling is claimed to be driven by emotion. Its key function is a meeting of hearts: a resonance in the recipient(s) of the story narrator’s emotion toward the story events (cf. Stivers, 2008). Insights into how emotional resonance is achieved in storytelling are beginning to flow from Physiological Interaction Research (e.g., Peräkylä et al., 2015). This paper aims to contribute to this line of inquiry. Its focus is on the role of *gestures* in emotion expression and emotion resonance in storytelling. Gestures are a core feature of face-to-face language use (Holler and Levinson, 2019; Kendon, 2004; Özyürek, 2017; Vigliocco et al., 2014), forming part of a multimodal Gestalt (Holler and Levinson, 2019; Trujillo and Holler, 2023) and providing both semantic and pragmatic meaning to the utterance (Kendon, 2017; Özyürek, 2014). What is more, gestures are an important tool in a story narrator’s toolkit for engendering resonance as gestures can also express emotions (e.g., Goodwin et al., 2012, p. 16; Selting, 2010, 2012; Couper-Kuhlen, 2012), and even influence memory and information uptake when paired with emotionally salient speech (Asalıoğlu and Göksun, 2023; Guilbert et al., 2021; Levy and Kelly, 2020). What is more, the performance of the same gesture can be varied along multiple kinematic dimensions such as speed, force, and size, thus creating an infinite number of expressive effects (cf. Dael et al., 2013). Such kinematic modulation of gesture has also been linked to the expression of different social intentions (Peeters et al., 2015; Trujillo et al., 2018), although its effect on emotional resonance is not yet known.

**Figure 1:**
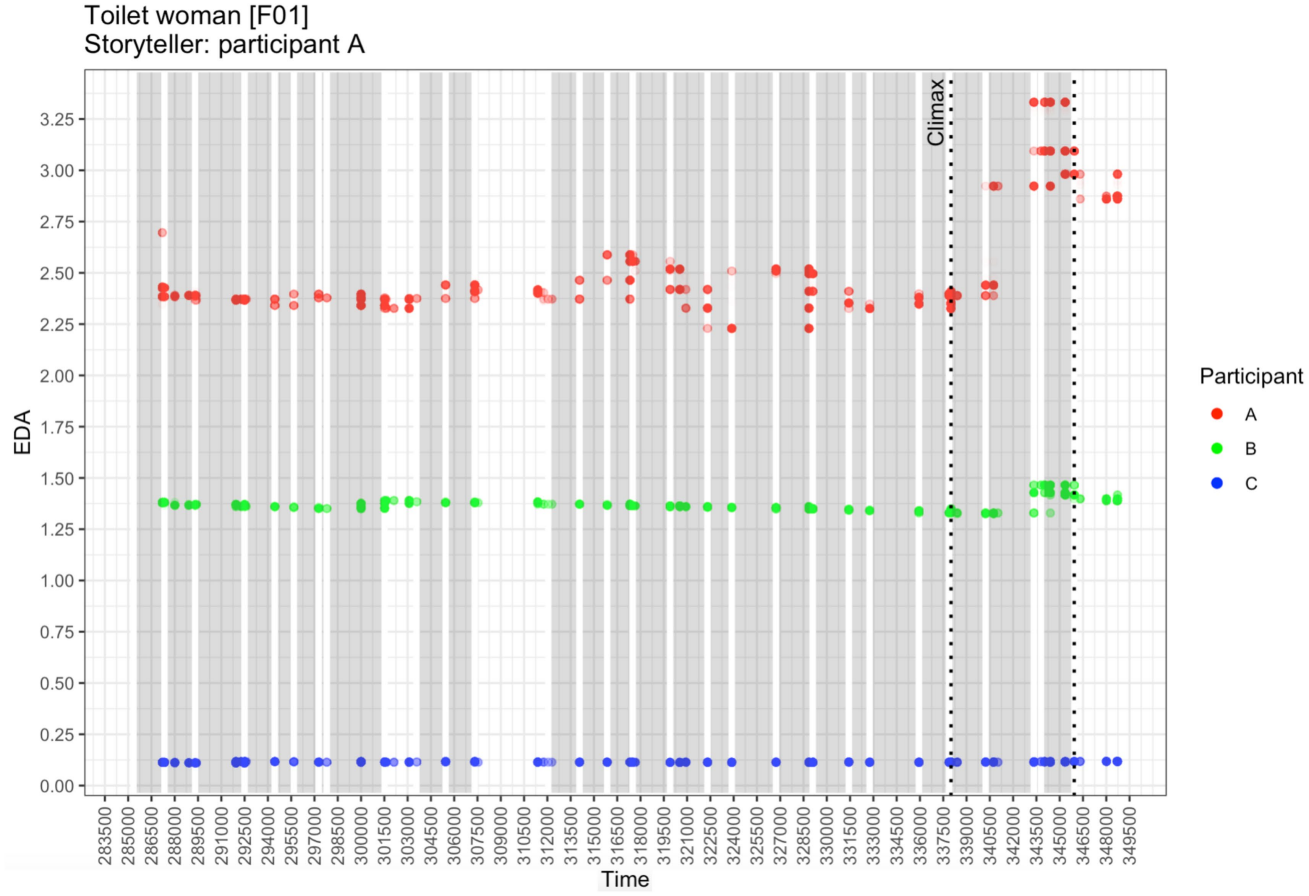
EDA responses in story “Toilet woman” by participant; gray rectangles demarcate the extensions of the story narrator’s gestures. Colored dots represent observed EDA measurements (indicated along the y-axis). Time is given along the x-axis, in milliseconds from the beginning of the recording.

To illustrate the interplay of gestures by a story narrator and the participants’ EDA responses, consider Extract 1 and Figure 1. The extract showcases a conversational storytelling by speaker A to two female story recipients, in which she expresses her distriss at frequently being misrecognized as a man. Her storytelling is accompanied by a large number of gestures; for space considerations only the three co-climax gestures are given below the simultaneous speech in Extract 1:

**Figure:**
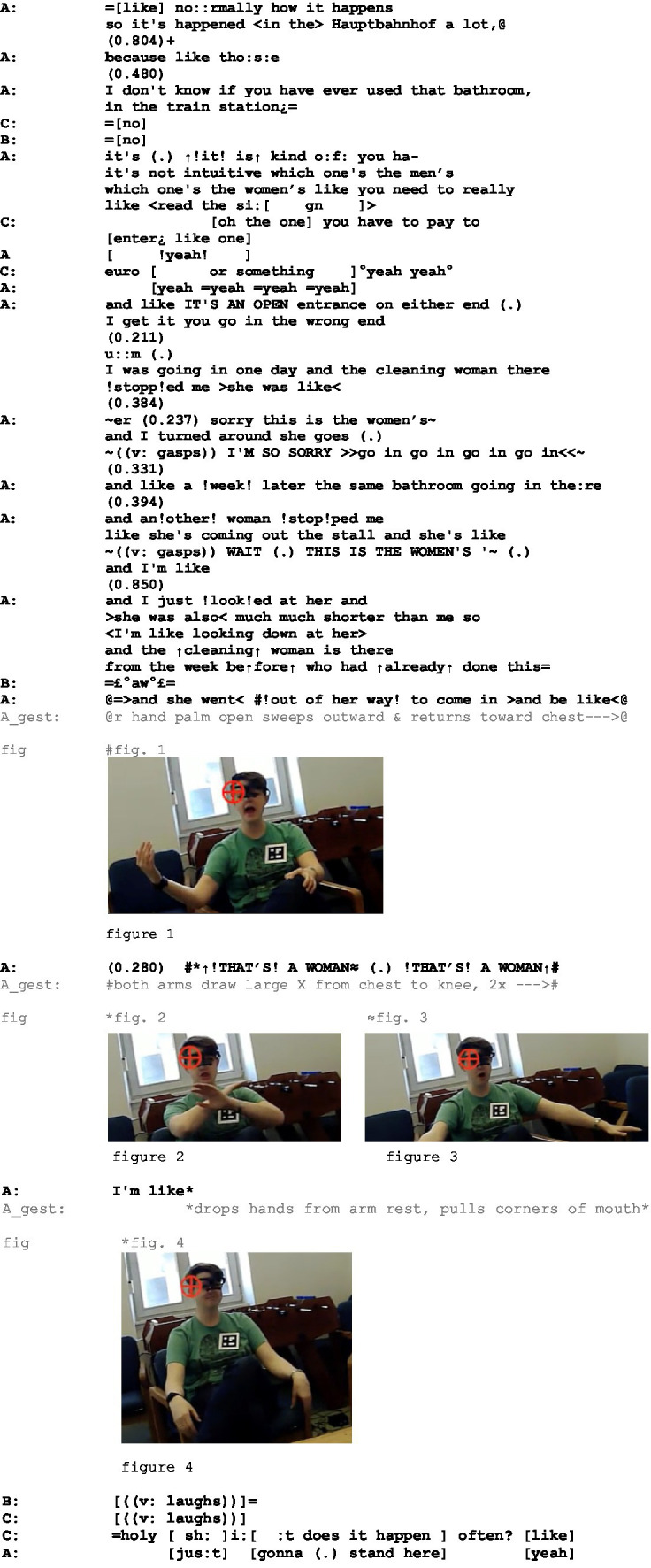


**Figure 2:**
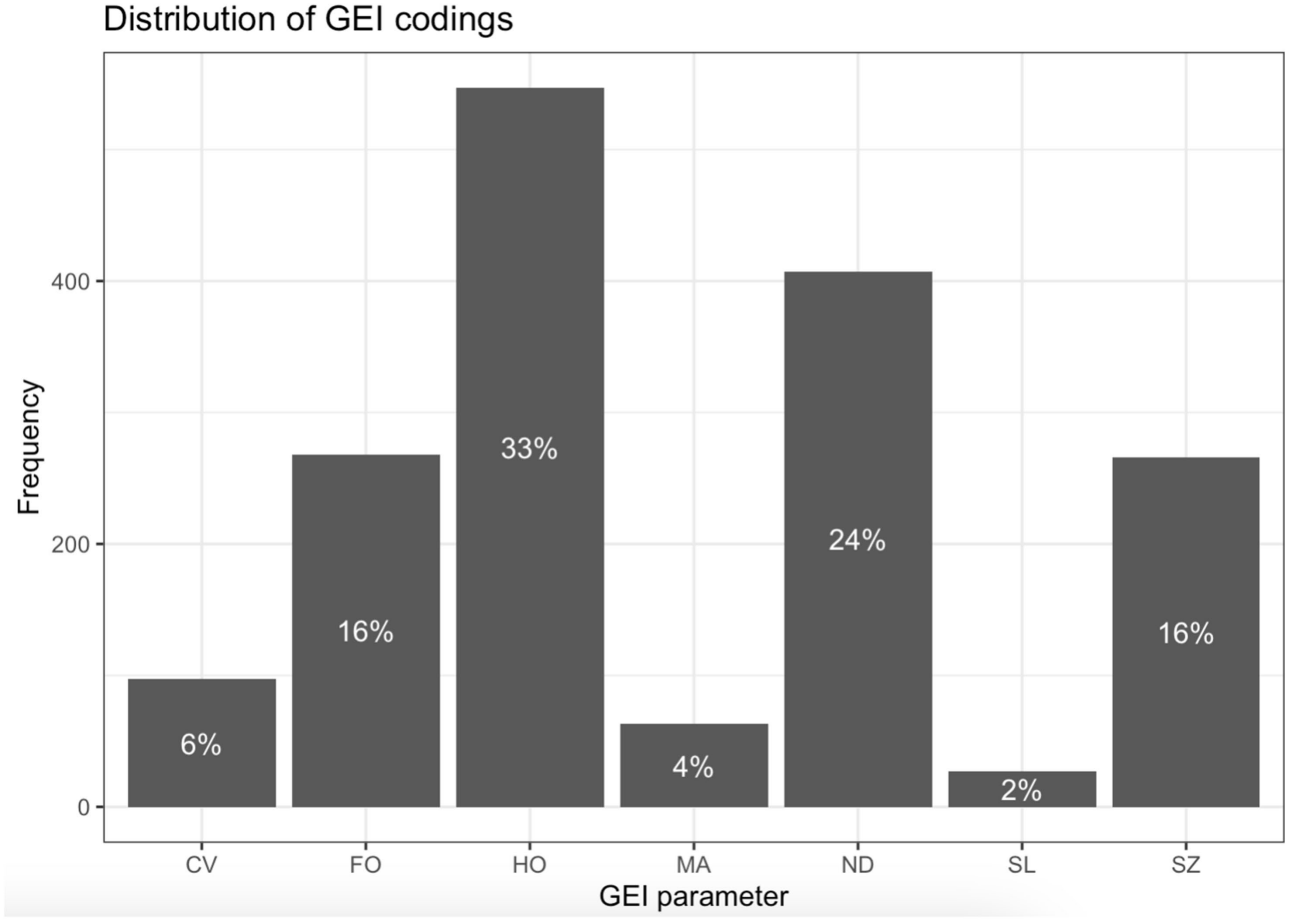
Frequency of Gesture Expressivity Index (GEI) features where coding was “yes”. The seven GEI parameters are given along the x-axis. The height of the bars indicates the percentage out of all gestures where a given parameter was coded as having a particular feature.

**Figure 3:**
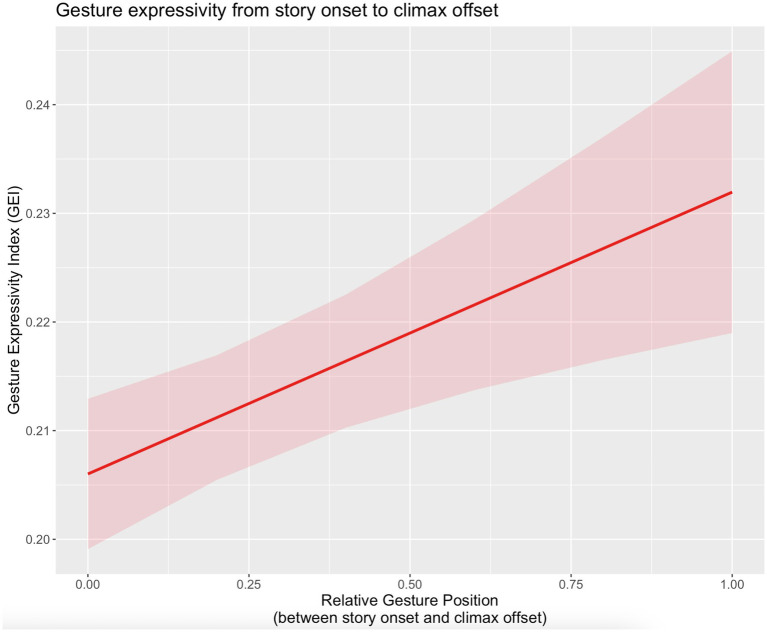
Linear mixed-effects regression model for gesture expressivity (*G_expressivity*) in storytellings (from story onset to climax offset); *G_position_rel*: relative positions of gestures in storytellings (values between 0 and 1).

**Figure 4:**
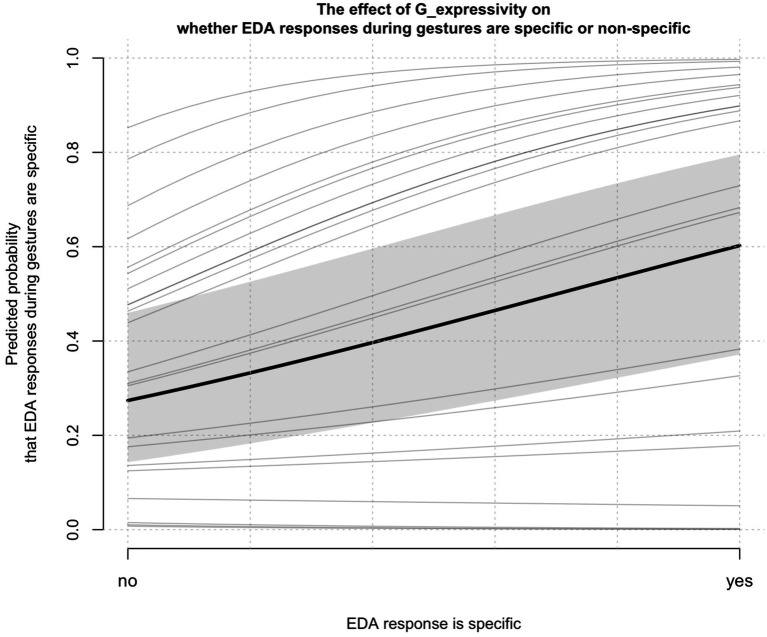
The effect of gesture expressivity (*G_expressivity*) on whether EDA responses are specific or not (*EDA_G_specific_responses_binary*); thin gray lines indicate individual participants.

The story revolves around the protagonist, speaker A, needing to use the same public bathroom twice and each time being denied entry to the women’s bathroom by a cleaning lady. While the first time around the misunderstanding is resolved by the cleaning woman recognizing and admitting her mistake (line 28), speaker A is again denied entry a week later by another cleaning lady. This time the misunderstanding is only resolved when the cleaning lady *from the week before* (line 41) intervenes on A’s behalf by loudly proclaiming ↑*!THAT*’*S! A WOMAN (.) [!THAT*’*S! A WOMAN*↑ (line 44). During this constructed dialog, A performs a dramatic character-viewpoint gesture twice: a large X with both arms by which the cleaning lady indicates that denying A entrance to the women’s bathroom is wrong (cf. embedded figures 2, 3). In line 45, A returns to her own character-viewpoint to depict her ensuing frustration and exhaustion: after she introduces constructed dialog with I’m like she silently drops her hands from the arm rest and pulls the corners of her mouth (cf. embedded figure 4).

The story recipients display sympathy with A: speaker B utters *£°aw°£* in a low smiley voice already in pre-climax position (line 42), while speaker C responds with a lengthened *= holy [sh:]i:[:t* in post-climax position (line 50).

The storytelling is performed with considerable gestural effort; altogether 25 gestures were annotated. Does the effort have any measurable effect on the recipients emotionally? Figure 1 depicts the EDA responses of all three participants during the telling.

As shown in Figure 1, in pre-climax positions, only the story narrator seems to experience changes in EDA, whereas the two recipients’ EDA responses remain largely flat. This changes during the climax: not only are there huge increases in EDA in the story narrator A, but there is a clear simultaneous hike in EDA in recipient B, specifically during the third in-climax gesture; in recipient C, however, EDA does not change at all.

As far as this storytelling is concerned, the story narrator and recipient B seem to resonate emotionally: their EDA responses peak at the same time, roughly in synchrony with the story narrator’s gestures.

The overarching goal in this paper is to examine whether the resonances that can be observed between participants in conversational storytelling interaction are an effect of the expressivity of the story narrator’s gestures. Early pioneering found that different emotions are expressed through different parameters of bodily movement and posture (Wallbott, 1998), and that bodily movement can express the intensity of emotions (Ekman and Friesen, 1974). Here, we focus on expressivity of co-speech gestures, using movement features that have been shown to be important indicators of expressivity. Specifically, the current measure of gesture expressivity captures similar dynamic aspects of gesture as previous work interested in quantifying expressivity, for example in the gesture synthesis literature, that independently assess features such as spatial and temporal extent, power, and fluidity (Hartmann et al., 2005; Pelachaud, 2009). Visual annotation of these features has similarly been applied to analyzing expressivity of observed gestures (Chafai et al., 2006; Kipp et al., 2007). The quantification of gesture expressivity used here captures many of these same kinematic features (i.e., size, force, hold-phases, bodily co-articulation, nucleus duration), but also includes dialogically relevant non-kinematic features (i.e., character-viewpoint, silence during gesture) and aggregates them into a singular value. This allows a more straightforward assessment of the role of gesture expressivity that accounts for both kinematic parameters as well as characteristics related to the gesture’s embedding in the ongoing multimodal narrative. Clearly, emotional resonance in conversational storytelling can result from factors and their interactions that are not part of or related to gesture performance as such but originate in the story narrator’s verbal performance and/or the design of the storytelling situation. Therefore, while we focus on the potential effect of gesture expressivity on emotional resonance we will approach the effect multi-factorially, by considering a large number of potentially contributing factors besides gesture expressivity.

As a first step to understanding the role of gestures in emotional resonance of conversational storytelling, we also assessed whether gesture expressivity follows the same “climacto-telic” (Georgakopoulou, 1997; Labov, 1972) structure that has been described for the oral component of storytelling. Climacto-telic refers to the gradual build-up of “tension” (Longacre, 1983) to be released only at climax. Initial observations gained from small-scale empirical analyses suggest that story narrators advance-project the story climax using an orchestrated crescendo of expressive multimodal means including constructed dialog, pitch, intensity, gaze alternation, and gestures (Mayes, 1990; Holt, 2007; Goodwin, 1984; Rühlemann, 2019; Rühlemann et al., 2019; Rühlemann, 2022).

Specifically, the paper asks three questions: Does story narrators’ gesture expressivity increase from story onset to climax offset (RQ #1)? Does gesture expressivity predict specific EDA responses in story participants (RQ #2)? How important is the contribution of gesture expressivity to emotional resonance compared to the contribution of other predictors of resonance (RQ #3)?

## 2 Methods

### 2.1 The FreMIC corpus

The data underlying the analyses in this paper come from the Freiburg Multimodal Interaction Corpus (FreMIC; cf. Rühlemann and Ptak, 2023). FreMIC is a multimodal corpus of naturalistic conversation in English, which is, at the time of writing, still under construction. All conversations were annotated and transcribed in ELAN (Wittenburg et al., 2006). The transcriptions follow conversation-analytic conventions (e.g., Jefferson, 2004) to render verbal content and interactionally relevant details of sequencing (e.g., overlap, latching), temporal aspects (pauses, acceleration/deceleration), phonological aspects (e.g., intensity, pitch, stretching, truncation, voice quality), and laughter.

### 2.2 Participants

Fourty-one individual participants were recruited to contribute to one or more of the 38 recorded conversations (total run time 30 h). The participants were mainly students at Albert-Ludwigs-University Freiburg as well as their friends and relatives [17 male, 21 female, 3 diverse/NA; mean age = 26 years (SD = 5.7 years)]. Most participants’ (*n* = 38) first language was English. All participants had normal or corrected to normal vision and hearing. Before the start of the recording, participants gave their informed consent about the use of the recorded data, stating their individual choices as to which of their data can be used and for what specific purposes. They received a compensation of €15 for their participation.

### 2.3 Procedure

Recordings were made in dyadic and triadic settings using one room camera and one centrally placed scene microphone. Seated in an F-formation (Kendon, 1973) participants were able to establish eye contact, hear each other clearly, and engage in nonverbal cues. Participants in dyads were seated vis-à-vis each other, with the room camera capturing both participants from the side. Participants in triads were seated in an equilateral triangle, with the room camera frontally capturing one of the participants and the other two from the side. The participants were told they were free to talk about anything for about 30–45 min until the recording would be stopped.

Participants wore Ergoneers eyetracking devices (Dikablis Glasses 3), which recorded the visual field of each participant plus the direction of participants’ gazes. Participants wore also Empatica wrist watches, which recorded a wealth of psycho-physiological data, including Electrodermal Activity (EDA), the measurement of central interest in this study. The wrist watches’ sampling frequency for EDA measurements is 4 Hz within a range of 0.01–100 μSiemens. Due to malfunction of the Empatica wrist watches, only nine recordings produced EDA data.

With the watches being placed at the wrists, EDA is measured in close proximity to the palms, where the highest concentration of eccrine sweat glands is found. The sweat produced by these glands is emotion-evoked (Dawson et al., 2000, p. 202) rather than thermo-regulatory (Bailey, 2017, p. 3; cf. also Scherer, 2005; Bradley and Lang, 2007), making palm-near EDA measurements a reliable indicator of emotional arousal (Peräkylä et al., 2015). Arousal is defined as the intensifying excitation of the sympathetic nervous system associated with emotion (Dael et al., 2013, p. 644; Peräkylä et al., 2015, p. 302). Heightened arousal results in increased EDA while emotional unaffectedness correlates with decreases in EDA. Being controlled by the sympathetic nervous system neither process can be influenced volitionally.

The focus in the present analysis is on phasic EDA representing “transient, wave-like changes which may be elicited by external stimuli or may be "spontaneous,” i.e., elicited by internal events” (Lykken and Venables, 1971, p. 657). Given the overall aim to examine the possible effect of gesture expressivity on emotional resonance, the phases during which all participants’ EDA is examined are the story narrator’s gestures (plus an additional time window to account for response latency; cf. Section 2.4.4).^1^

### 2.4 Data pre-processing

#### 2.4.1 Story selection

53 conversational storytellings were selected for this analysis. Given the scarcity of recordings with EDA measurements (cf. Section 2.3), the selection criteria for stories were relatively broad. The only must-have criteria were (i) anterior situation (the story events happened in the past rather than in the future or in an imaginary world, cf. Norrick, 2000) (ii) involving at least one a-then-b relation (i.e., the temporal sequencing of at least two narrative events; cf. Labov and Waletzky, 1967),^2^ (iii) extension (all stories except one are longer than half a minute, thereby excluding so-called “small stories,” cf. Bamberg, 2004), and (iv) the use of constructed dialog (also referred to as “quotes,” “direct speech” or “enactments”; cf. Labov, 1972).

Story climaxes were identified as those story events that semantically matched the emotion expressed at story onset. For example, the climax to a story billed as “sad” was the story’s sad(dest) event, the fun(niest) event was coded as the climax in a “funny” story. Another identification criterion was the occurrence of direct speech; this criterion relies on the widely accepted notion that direct speech clusters at story climaxes (cf. Labov, 1972; Li, 1986; Mathis and Yule, 1994; Mayes, 1990; Norrick, 2000; Clift and Holt, 2007; Rühlemann, 2013). Another criterion used to identify a story’s climax was “texturing” (Goodwin, 1984), that is, variations in the story narrator’s paralinguistic prosody such as raised pitch and increased intensity. Finally, given that storytellings constitute activities centered essentially around stance and emotion, climaxes are characterized by recipients mirroring the story narrator’s stance/emotion displayed earlier (implicitly or explicitly). Verbally, that mirroring is achieved through the use of tokens of *affiliation* displaying the recipient’s stance, including, for example, assessments such as *wow* (Goodwin, 1986), head nods (Stivers, 2008) or laughter (Sacks, 1978).^3^

#### 2.4.2 Story annotation

The stories were rated for a large number of variables that may potentially impact emotion arousal. These include (i) *Protagonist* (whether the story’s protagonist is the story narrator or a non-present third person; cf. Ochs and Capps, 2001), (ii) *Recency* (whether the story events occurred far in the past or were occurring at or close to storytelling time), (iii) *Group_composition* (whether groups were all-female, all-male, or mixed), and (iv) *Group_size* (whether the storytelling setting was dyadic or triadic).

The variables *Protagonist* and *Recency* were considered potentially impactful for emotional resonance as they are components of *relevance*, a key dimension of emotion, as “in order for a particular object or event to elicit an emotion, that object or event needs to be […] relevant to the person in whom that emotion is elicited” (Wharton et al., 2021, p. 260). Indeed, emotions can be seen as “relevance detectors” (Scherer, 2005, p. 701). The factor *Group_size* is included somewhat tentatively based on the recent finding that response times are faster in triads than dyads (Holler et al., 2021) due to competition. While, obviously, competition in triads is not emotion *per se*, competition may nonetheless contribute to heightened emotion arousal. *Group_composition* was included as predictor to capture potential effects of gender. In Doherty (1997), for example, women were highly significantly more susceptible to emotion contagion than men (but see Eisenberg and Lennon’s, 1983 large meta-study, in which females did not exhibit more empathy than males when physiological measures were used to index empathy).

The variable *Sentiment* was calculated to account for the emotional impact of individual words uttered in each interpausal unit (IPU). Sentiment analysis was performed on individual words using the Python package *Vader* (Hutto and Gilbert, 2014), providing the associated positive, negative, and composite (combined) sentiment scores for each transcribed word. The composite sentiment score was taken, which reflects the composite of both positive and negative scores for each given word. The mean composite score was calculated for each IPU, and each gesture (described below) was assigned the *Sentiment* score of the IPU with which it occurred.

#### 2.4.3 Gesture annotation

1,021 gestures as well as their gesture phases (Kendon, 2004) were identified and annotated in ELAN by multiple raters. The gestures were further coded in ELAN for seven gesture-dynamic parameters: (i) Size (*SZ*; Dael et al., 2013), (ii) Force (*FO*; Dael et al., 2013), (iii) Character view-point (*CV*; McNeill, 1992), (iv) Silence during gesture (*SL*; Hsu et al., 2021, p. 1; Kendon, 2004, p. 147; Siddle, 1991, p. 247), (v) Presence of hold phase (*HO*; Beattie, 2016, p. 129; Gullberg and Holmquist, 2002), (vi) Co-articulation with other bodily organs (*MA*; Dael et al., 2013; Rühlemann, 2022) and (vii) Nucleus duration (*ND*; Kendon, 2004).

In judging gesture size (SZ), lateral and forward movements were distinguished. Size in lateral movements was coded based on McNeill’s (1992) gesture space schema. A gesture was considered sizable if it crossed at least two major lines in the gesture space schema; e.g., from CENTER-CENTER to PERIPHERY, or from EXTREME PERIPHERY to CENTER. If the onset of a gesture was not at the “normal” rest position (i.e., in the speaker’s lap or on the arm rest) but at some other point in the gesture space, that onset was taken as the starting point of the gesture’s trajectory and the count of how many major boundaries it crossed started from there. For example, if the gesture’s onset was in the right EXTREME PERIPHERY and moved back to PERIPHERY, one single major boundary is crossed and the movement was considered not sizable. Gesture size can also become expansive if the hands’ and arms’ orientation is away from the gesturer’s body into the space in front of them, i.e., if they extend their hands toward the interlocutor. Forward gestures were coded sizable only if the speaker extended her arms beyond a 45° degree angle.

Gestures were coded forceful (FO) based on the requirement that the movement requires muscular effort. To gage whether muscular effort was involved, annotators physically reenacted the gesture. Also, a diagnostic of a forceful gesture is the whiplash effect, i.e., when the hand slightly bounces back from the gesture’s endpoint. Gesture force undoubtedly enters into a number of interactions with other dynamic parameters. Clearly, the more sizable a gesture the more muscular effort it will involve. Also, extended holds (especially of sizable gestures) likely require muscular effort, as do gestures that are carried out fast, again especially if they are sizable. The fact that FO (force) saw the least interrater agreement (cf. Table 1) is therefore not surprising.

**Table 1:** Interrater agreement on gesture expressivity index (GEI) parameters (based on 227 gestures, or 22%, out of 1,021 gestures in total); the GEI parameters *Hold phase* (*HO*), *MA* (Co-articulation with other bodily organs), and Nucleus duration (ND) were calculated directly from their annotations in ELAN.

GEI parameter	Agreement %	Interrater reliability (G)
CV	94.27	0.89
FO	78.41	0.57
SL	97.36	0.95
SZ	86.78	0.74

Gestures were coded as character-viewpoint gestures (CV) if they were carried out as if the gesturer slipped into the role of the character; alternatively, the gesture is performed from the gesturer’s own perspective, as if observed by them (cf. McNeill, 1992; Beattie, 2016). While character-viewpoint gestures are often representational gestures, in most co-quote gestures the movements carried out by the gesturer were the character’s movements not the gesturer’s regardless of type of gesture. The inclusion of CV among the (potentially) expressive gesture features is based on the observations that (i) character-viewpoint gestures are more effective for communicative purposes than observer viewpoint gestures (Beattie, 2016), (ii) they represent “demonstrations” rather than “descriptions” (Clark and Gerrig, 1990) allowing the storytelling recipients to immediately and immersively see and experience the displayed emotions without the observer’s intermediary perspective separating the audience from them, and (iii), in our data, they overwhelmingly occur within direct quotation, a discursive practice that facilitates heightened multimodal activation in speakers (e.g., Blackwell et al., 2015; Stec et al., 2016; Soulaimani, 2018).

Silent gestures (SL), alternatively referred to as “speech-embedded non-verbal depictions” (Hsu et al., 2021), are gestures that communicate meaning “iconically, non-verbally, and without simultaneously co-occurring speech” (Hsu et al., 2021, p. 1). With the (default) verbal channel muted, the burden of information is completely shifted to bodily conduct (cf. Levinson and Holler, 2014, p. 1). This shift makes silent gestures particularly expressive: they are “foregrounded” and “exhibited” (Kendon, 2004, p. 147).

Actively attending to them is prerequisite for the recipient’s understanding. Moreover, given that the occurrence of speech is expected, its absence will not only be noticeable but also emotionally relevant as the omission of an expected stimulus has been shown to increase EDA response (Siddle, 1991, p. 247).

Subsuming the presence of a hold phase (HO) under expressive gesture dynamics draws on the absence of movement, which is assumed to gain saliency considering the lack of progressivity manifested in the hold. Given the preference for progressivity (Stivers and Robinson, 2006; Schegloff, 1997), which we assume extends to a preference for progressivity in bodily conduct, the uninterrupted execution of a gesture can be seen as preferred, aligned with the default expectation of progressive movement, whereas the interrupted execution as occurring during a hold phase will be seen as disaligned with the default expectation of progressive movement and hence dispreferred. As a dispreferred, the gesture hold, just as a “hold” during speech, “will be examined for its import, for what understanding should be accorded it” (Schegloff, 1997, p. 15) and is therefore likely to raise attention and add to the saliency of the gesture. Gesture holds have also been linked to saliency in silent gesture paradigms (Trujillo et al., 2018), where longer hold-times are associated with better gesture recognition (Trujillo et al., 2020). Also, while the overwhelming majority of gestures are not gaze-fixated, i.e., not taken into the foveal vision, but still processed based on information drawn from the parafoveal or peripheral vision (cf. Beattie, 2016, p. 129), those gestures that contain a hold phase, i.e., a momentary cessation in the movement of the gesture, reliably attract higher levels of fixation (Gullberg and Holmquist, 2002). Beattie argues that “[d]uring ‘holds’, the movement of a gesture comes to a stop and thus the peripheral vision is no longer sufficient for obtaining information from that gesture, thus necessitating a degree of fixture” (Beattie, 2016, p. 131).

The annotations were implemented using a binary scale (yes/no) and aggregated in the Gesture Expressivity Index (GEI). The Index computes for each gesture an average value across all yes/no ratings; the Index values are stored in the variable *G_expressivity*, one of the key variables in the models. Note that this GEI value is thus a combination of kinematic salience values (4 features): SZ, FO, HO, and ND), multimodal coarticulation (two features, SL and MA), and one capturing a broader narrative embodiment (CV). There is, therefore, a somewhat heavier weighting of kinematic features in the calculation of GEI compared to other features. To date, there is no research indicating the actual weight of each of these features in predicting how “expressive” a gesture is perceived to be. Additionally, there was the possibility that character viewpoint gestures, which are defined more broadly (i.e., not based on more fine-grained movement parameters) would also be more kinematically salient. As a basic check, we conducted simple chi-square tests to assess whether there was evidence for the character viewpoint gestures being more likely to be larger, more forceful, or more likely to have a hold-phase than non-character-viewpoint gestures, and we did not find evidence for this to be the case (*p*-values from 0.635 to 0.921). For the purpose of this study, we therefore take the entire set of seven features as being relatively independent and equal in importance. The distribution of the GEI parameter codings is shown in Figure 2.

As shown in Figure 2, a third of all gestures had a hold phase (HO) and in roughly a quarter of them the duration of the nucleus was longer than the average nucleus in the respective story (ND); on the other hand, gestures were rarely coded as character viewpoint gestures (CV, 6%), gestures performed together with other bodily articulators (MA, 4%) and silent gestures (SL, 2%). Across all gestures, GEI ranged from 0 to 0.857, with a median value of 0.286. This indicates that most gestures (79%) showed two or fewer GEI features.

Interrater agreement for the coding of the GEI parameters was tested on c. 22% of all 1,021 gestures. Interrater agreement percentages ranged between 78% for Force (*FO*) and 97% for Silent gesture (*SL*). To calculate interrater reliability we used Holley and Guilford (1964) *G* index; the G values are reported in Table 1. We chose to use *G* as it provides a more robust measure of reliability than Cohen’s Kappa when the rating distributions are skewed (Silveira and Siqueira, 2023; Xu and Lorber, 2014). In the case of our GEI parameters, there was a heavy skew toward “negative” ratings, meaning that most gestures were coded as *not* having a particular parameter. CV and SL ratings showed near perfect reliability, SZ showed substantial reliability, and FO showed moderate reliability. While the lower *G* index for FO indicates that gesture force is a more difficult parameter to reliably code, the lower *G* is likely also due to the skewed coding. Specifically, 78% of the reliability-coded gestures received a *no* coding from at least one rater, with a true negative rate of 56%.

The parameters HO (hold), SL (silent gesture), and multiple articulators (MA) were extracted from the ELAN gesture and gesture phase annotations and were therefore not tested for interrater reliability. For example, whether a gesture was performed together with other bodily articulators was read off the gesture descriptions that indicated all articulators used. For example, the last in-climax gesture in extract (1) has this description: *((112_m & f: both h rested on arm rest slightly drop, pulls corners of mouth))*. Here, the initials “m” and “f” refer to manual (hand) and face as the articulating organs.

Further, based on FreMIC’s existing annotation of quotes (alternatively referred to as direct speech [e.g., Labov, 1972), constructed dialog (e.g., Tannen, 1986), or) and enactments (e.g., Holt, 2007)] and using a fuzzy assignment procedure which allowed for a durational “distance” of 1.5 s. of the respective start times, the gestures were examined for whether they co-occurred with a quote (variable *G_quote*). Instances of direct speech are likely to impact emotional resonance not only as they thrive in storytellings (Rühlemann, 2013; Stec et al., 2016) but also because they facilitate heightened activation in the speaker’s vocal and bodily channels (e.g., Blackwell et al., 2015; Stec et al., 2016; Soulaimani, 2018) and because they are frequently mimicry (e.g., Mathis and Yule, 1994; Stenström et al., 2002, p. 112; Halliday and Matthiessen, 2004, p. 447), “a caricatured re-presentation” (Culpeper, 2011, p. 161) or “echo” of anterior discourse, “reflect[ing] the negative attitude of the echoer toward the echoed person” (Culpeper, 2011, p. 165).

The total number of stories the present analyses are based on 53 stories collected in nine recordings (total run time 7.55 h), with 1,021 gestures by the story narrators, and 14 distinct participants (Table 2).

**Table 2:** Sociodemographics of FreMIC participants to this study.

Sex	*N*	Mean age	Age range	SD	L1
Female	5	26	22–31	3.39	English: 4; other: 1
Male	8	31	24–49	9.08	English: 8
NA/Diverse	1	27	27	*NA*	English: 1

The familiarity levels between the participants in the recordings were mixed throughout. While in some triadic recordings either siblings or romantic partners participated (with high familiarity), the third subject was always either a stranger (low familiarity) or an acquaintance (medium familiarity), while the dyadic conversations were invariably between friends or acquaintances. Applying a three-level distinction (low, mixed, or high familiarity) would have resulted in a single value, “mixed.” We therefore decided not to use familiarity as a predictor in our models.

#### 2.4.4 EDA pre-processing

To account for response latency (typically between 1 and 3 s; Dawson et al., 2000, p. 206), EDA responses were measured during the duration of the gesture as well as 1.5 s post-gesture. Further, EDA responses were classified as *specific* (i.e., as indexing a stimulus-related emotional response) if they were larger than 0.05 μSiemens. Finally, *resonating gestures* were identified, on the condition that they exhibited *concurrent* specific EDA responses by two or more participants. The result is a binary variable *EDA_G_resonance*, which represents the dependent variable in the Random Forest model (see below).

### 2.5 Statistical analysis

RQ #1 was addressed using a mixed-effects model. In order to handle the large variance in the number of gestures used in the stortyellings (the range is 4–91 gestures; mean = 21.6; median = 15.5), a relative positional measure *G_position_rel was* computed for each gesture in each story assigning as many equi-distanced values between 0 and 1 as there are gestures in the storytelling (e.g., the relative positions of 4 gestures are 0, 0.25, 0.5, 0.75, and 1). The fixed effects in the model were *G_expressivity* (as the response variable) and *G_position_rel* (the independent variable); the random variable was ID, a combination of participant and recording ID.

To adress RQ #2, a second linear mixed-effects regression model was constructed, with *EDA_specific_response_binary* as the dependent and *G_expressivity* as the independent variable, as well as *Participant* and *Recording* modeled as random effects. If there were not issues with model fit, we modeled random slopes, rather than random intercepts.

For the mixed models described for RQ #1 and #2, statistical significance was determined using a log-likelihood test, comparing the full model as described above against a null model having the same structure but without the main independent variable of interest. For RQ #1, the null model did not include *G_position_rel*, while for RQ #2, the null model did not include *G_expressivity*. We report conditional pseudo-R^2^, as calculated by the *MuMIn* R package (Bartoń, 2009) as a measure of effect size.

RQ #3 requires a different statistical approach, as it specifically addresses the magnitude of the influence of *G_expressivity* and, respectively, the gesture dynamics that feed into it, on the outcome variable *EDA_G_resonance* relative to the magnitudes of other potentially impactful predictors, most of which can be assumed to be highly collinear. The method warranted by this type of research scenario is a Random Forest model. Random Forests are able to handle collinear features effectively, based on vertical sampling of variables (feature subsampling), horizontal sampling of data (bootstrapped sampling), and random decision tree splitting based on a single predictor at a time thus mitigating the influence of collinearity not only within individual trees but also across trees. Another advantage of Random Forests is that they indicate relative variable importances (cf. Tagliamonte and Baayen, 2012; Gries, 2021). The Random Forest built here comprises 1,500 trees (ntree = 1,500) and three randomly preselected predictors at each split (mtry = 3).

To investigate the effect on emotional resonance of potential interactions between predictors, Individual Conditional Expectation (ICE) plots (Goldstein et al., 2015) were inspected. These plots visualize how changes in a single predictor affect the predicted response of a model *for each individual instance*. Each line in an ICE plot represents the prediction for a single case of the response (here, gesture-related emotional resonance) as the predictors of interest change while keeping all other predictors constant. ICE plots are particularly useful for identifying interactions between the response and the predictors in the model.

## 3 Results

### 3.1 Does story narrators’ gesture expressivity increase from story onset to climax offset (RQ #1)?

We found that gesture position (*G_position_rel*) was associated with *G_expressivity* [*χ*^2^(3) = 20.902, *p <* 0.001, with gestures that occurred later in a story showing higher *G_expressivity* values (see Table 3). Checking the random slope coefficients for each speaker revealed that 77% of speakers showed this positive association. Conditional pseudo-R^2^ for this model was 0.036.

**Table 3:** Output of linear mixed-effects regression model on *G_expressivity* in storytellings.

Fixed effects	*β*	*SE*	*df*	*t*	*p*	*Sig*
(Intercept)	2.048e-01	6.484e-02	7.965 e-02	31.585	<0.001	***
G_position_rel	2.786e-02	1.585e-02	1.825 + 03	1.785	0.095	

Formula: G_expressivity ~ G_position_rel + (1 | ID).

### 3.2 Does gesture expressivity predict specific EDA responses in story participants (RQ #2)?

We found that the probability of specific EDA responses was positively associated with *G_expressivity* [*χ*^2^(1) = 18.046, *p <* 0.001]. See Table 4 for an overview of model coefficients. Only random intercepts were included in this model due to singular fit when including random slopes. Note that while there is a large spread of intercepts across individual participants in Figure 3, indicating a large amount of variance across participants, nearly all of the fit lines show the same positive association. Conditional pseudo-R^2^ for this model was 0.464.

**Table 4:** Output of linear mixed-effects regression model on G_expressivity in storytellings.

Fixed effects	*β*	*SE*	*z*	*p*	*Sig*
(Intercept)	−0.9489	0.4183	−2.269	0.0233	*
G_expressivity	1.4314	0.3378	4.237	2.26e-05	***

Formula: EDA_G_specific_responses_binary ~ G_expressivity + (1 | ID).

### 3.3 How important is the contribution of gesture expressivity to emotional resonance compared to the contribution of other predictors of resonance (RQ #3)?

The Random Forest (*ntree* = 1,500, *mtry* = 3) that was constructed for emotional resonance (*EDA_G_resonance*) as outcome variable and the seven GEI parameters as well as six more variables as predictors (*G_quote*, *Sentiment*, *Protagonist*, *Group_compose*, *Group_size,* and *Recency*) exhibited a very good fit: according to a one-tailed exact binomial test, the model was significantly better than chance/baseline (*p* < 0.001), the (traditional) R^2^ was 0.876, and McFadden’s R^2^ scored an excellent 0.386.

**Figure 5:**
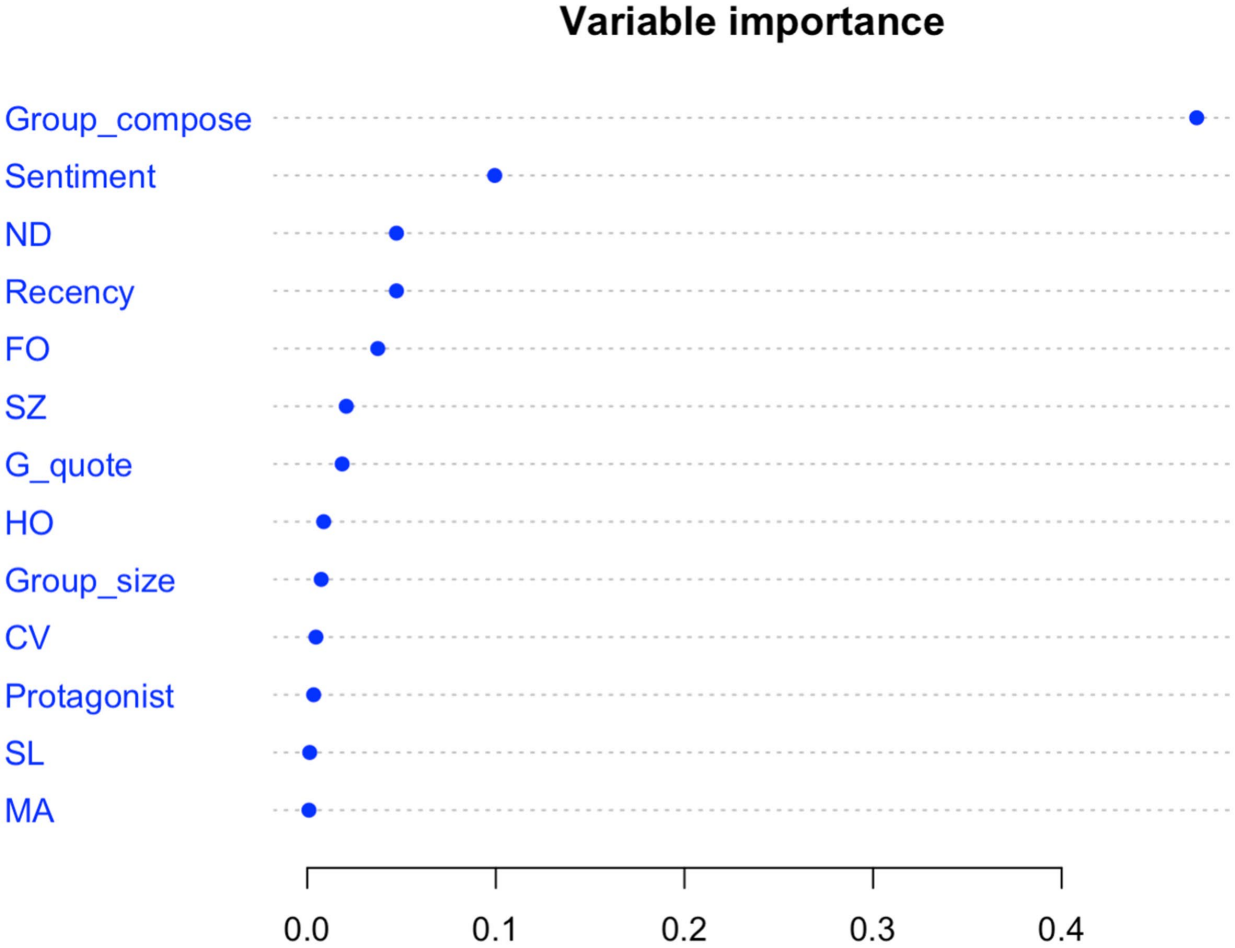
Overview of conditional variable importance in the Random Forest model predicting probability of specific EDA response. Individual predictor variables are given on the y-axis, while conditional variable importance is on the y-axis. Variable importance represents the mean decrease in accuracy of the model prediction when a given variable is removed.

All predictors were found to impact *EDA_G_resonance*. Analysis of variable importance showed *Group_composition* to be by far the most impactful predictor, followed by *Sentiment*, *ND* (nucleus duration), *Recency, FO* (gesture force), *SZ* (gesture size), *G_quote* (gesture is co-quote), *HO* (gesture includes hold phase), *Group_size, CV* (gesture is character viewpoint), *SL* (silence during gesture), *Protagonist*, and *MA* (multiple articulators). While all variables had positive importance scores (indicating they contribute positively to model accuracy) the scores for *SL* (0.0013) and *MA* (0.0009) are very small (likely a reflection of their rarity; cf. Section 2.4.3) (See Figure 5).

**Figure 6:**
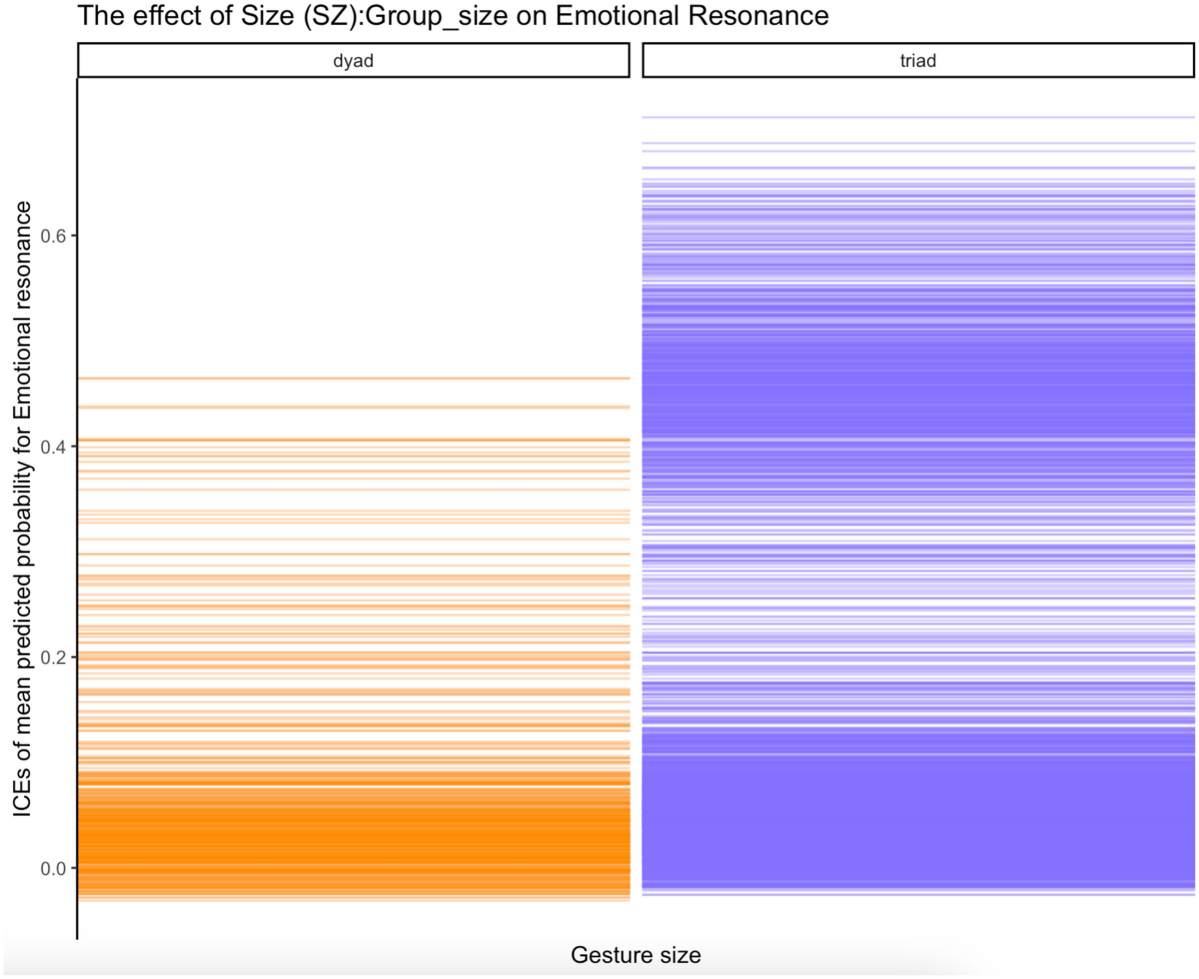
ICE plot of effect of interaction of Gesture size (*SZ*): Group size (*Group_size*) on Emotional resonance (*EDA_G_resonance*); values on the y-axis represent jittered means of predicted probabilities that emotional resonance is achieved (*EDA_G_resonance* = “yes”).

**Figure 7:**
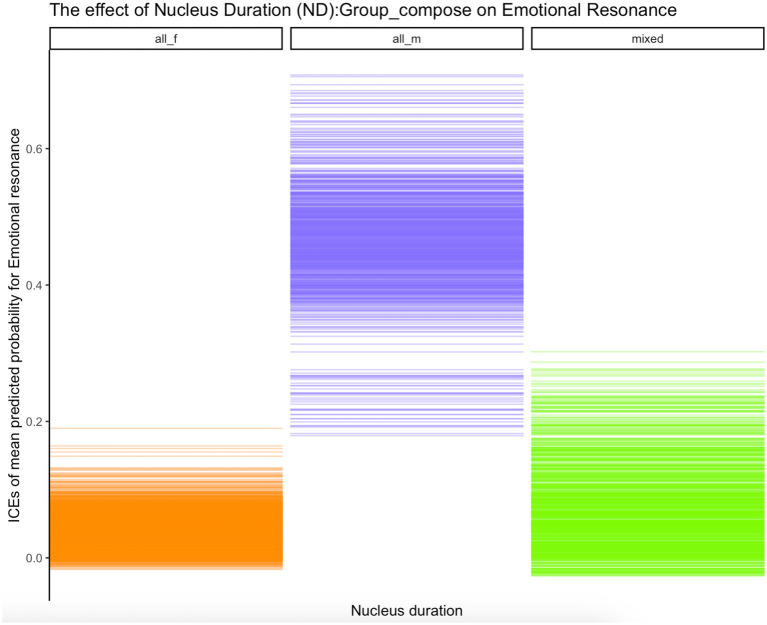
ICE plot of effect of interaction of Nucleus duration (*ND*):Group composition (*Group_compose*) on Emotional resonance (*EDA_G_resonance*); values on the y-axis represent jittered means of predicted probabilities that emotional resonance is achieved (*EDA_G_resonance* = “yes”).

Inspection of ICE plots strongly indicated combined effects of individual GEI parameters and other factors, including *Group_size* [the probabilities that gesture force (FO), size (SZ) and, respectively, nucleus duration (ND) impacts *EDA_G_resonance* were higher in triads] and *Group_compose* (the probabilities that these parameters impact *EDA_G_resonance* were much higher for all-men groups than for all-female and mixed groups). Figure 6 shows an ICE plot depicting the effect on emotional resonance of gesture size (*SZ*) interacting with group size (*Group_size*), while Figure 7 depicts the effect on emotional resonance of nucleus duration (*ND*) interacting with group composition (*Group_compose*). As can be seen from the plots, the effects of the two gesture kinematics (size and, respectively, nucleus duration) are much stronger for triads than for dyads on the one hand and for all-men groups than for all-female or mixed groups on the other. Note that these combined effects for group size and, respectively, group composition and gesture kinematics on emotional coupling were observed *consistently* across all seven gesture kinematics (FO, ND, SZ, HO, CV, MA, and SL).

## 4 Discussion

Overall, this study suggests that gesture expressivity increases over the course of a story and contributes to emotional resonance in conversational storytelling interaction.

Examination of RQ #1 demonstrated that story narrators’ gestures become more expressive from story onset to climax offset. As the relation of gesture expressivity with conversational storytelling progression^4^ has, to the best of our knowledge, not yet been examined statistically, this finding is the first of its kind. Gesture expressivity thus joins the group of expressive multimodal means, such as constructed dialog (e.g., Mayes, 1990), gaze alternation (Rühlemann et al., 2019) as well as intensity and pitch (Goodwin, 1984), that story narrators ratchet up to advance-project the imminent arrival at the story climax. Our finding adds further weight to the *Multimodal Crescendo Hypothesis* (Rühlemann, 2022), which posits that the story narrator’s multimodal effort is *synchronized* with the storytelling’s progression toward the Climax: “multimodal resources are deployed climactically such that they peak when the telling peaks reaching its key event, thus illuminating it brightly so that the event will be recognized as the key event at which displays of emotional contagion are relevant” (Rühlemann, 2022, p. 22). While this multimodal progression was present in most (77%) of storytellings, it should be noted that not all participants showed this same progression. This means that while there is evidence for a multimodal crescendo, not all speakers or stories will show this effect. Whether such differences are more speaker-specific or story-specific is an interesting avenue for future research, as this may be informative for how different types of stories utilize multimodal resources in different ways in order to build up to their climax, or whether speakers do not fully utilize visual signals to deliver their story. In the latter case, this would be a potentially fruitful avenue for investigating and/or training effective storytelling practices.

Addressing RQ #2, we found that increased gesture expressivity increases the probability of specific EDA responses (indicating emotion arousal) in the partcipants to the storytelling interaction. This finding is strong evidence that gestures in conversational storytelling can have an effect on the way the telling is *experienced* emotionally by the participants. Story narrators exploit the expressive claviature of gestures, skillfully varying and intensifying gesture kinematics to effectively change the way the recipients feel: far from merely listening to and comprehending what happened they get pulled into the story narrator’s emotional orbit, potentially allowing them to partake in their emotions. This is consistent with the idea that multimodal expression enhances perceived emotions (Kelly and Ngo Tran, 2023), as well as the large body of research suggesting that the display of emotion, as in gestures, stimulates reciprocal emotional response (cf. Keltner and Ekman, 2000 and references therein). It is also consistent with the observed tendency for people in interaction to continuously and non-consciously monitor and mimic the other’s emotional expression and to “synchronize [their] expressions, vocalizations, postures, and movements and, consequently, to converge emotionally” (Hatfield et al., 1994, p. 5; Doherty, 1997, p. 149). The emotional contagion we observe in conversational storytelling interaction is clear evidence that storytelling has far deeper functions than just updating others so they know what happened; in storytelling, gestures (and other expressive means) can effectively evoke emotional arousal in their recipients, allowing for a more holistic experience of the story. Given that empathy comprises both cognitive empathy, “the intellectual/imaginative apprehension of another’s mental state” (Lawrence et al., 2004, p. 911), and affective empathy playing out on the psycho-physiological level of emotion, the power of storytelling derives from the fact that it activates both dimensions of empathy. Affective empathy can be of two kinds, parallel and reactive. If the observer’s empathic response matches that of the observed (your joy becomes my joy), the empathy is parallel; if the observer’s response is complimentary (your distress becomes my compassion), the empathy is reactive. But, as noted, based on EDA only, we cannot distinguish kinds of empathy. Whether gestures evoke these *parallel or complimentary* emotional states in the recipients as conveyed or intended by the story narrator will require further research utilizing different methods.

Note, however, that specific EDA responses only indicate the arousal of emotion as such. They do not allow us to identify the kind of emotion the person is experiencing. To identify particular emotions, alternative psychophysiological metrics are required. For example, decelerating heart beat is indicative of sympathetic observers of other’s sadness/distress, while the heartbeat of an observer with a self-focused personal distress reaction accelerates (Eisenberg et al., 1989 and references therein; cf. also Keltner and Ekman, 2000; Ekman et al., 1983).

Focusing on RQ #3, the analysis also demonstrated that gesture expressivity and the gesture dynamics that contribute to it allow conversational storytelling participants to resonate emotionally with one another by experiencing *simultaneous* emotion arousal. Considering that emotions are basic in the sense that they may have evolved “for their adaptive value in dealing with fundamental life tasks” (Ekman, 1999, p. 46) such as loss, danger, achievement, or fulfillment, being moved emotionally when the other is moved emotionally is, then, to resonate vis-à-vis any such fundamental life task. Darwin (1872) suggested that by sharing emotional states, individuals faced with such life tasks can bond, warn each other of danger, coordinate group activities, and, ultimately, enhance their chances of survival. In support of this claim, for example, Dunbar et al. (2016) demonstrated that, due to activation of the endorphine system, watching tragic films together increased not only social bonding but also tolerance of pain, thus making people effectively more resilient. Taking this evolutionary perspective, we can also speculate that emotional resonance in conversational storytelling would support the sharing of advice and (life) strategies, in the form of stories, where recognizing the emotions of events would be important. Our results suggest that gestures can support this resonance, and kinematic modulation of these gestures further contributes to the emotional resonance.

However, the analysis also demonstrated that gesture expressivity and the contributing gesture dynamics are just one set of factors in a complex web of factors that are *together* co-responsible for whether or not conversational storytelling participants get into synch emotionally with one another. Based on the analysis of variable importance, the analysis even suggested that gesture expressivity may not be the most impactful factor. Other non-gesture-related factors include, for example, the gender composition of the group of participants (by far the most important factor to emerge from the Random Forest model), the intensity of sentiment expressed in the co-gesture speech, whether the story events can be considered relevant given their recency and the co-presence of the protagonist, whether the story is told in dyads or triads, and whether the gesture is a co-quote gesture (i.e., whether it is part of the delivery of constructed dialog). Including the sentiment intensity of co-gesture speech, and seeing that gesture kinematics are still important in the model, further demonstrates that the synchronized affective responses of story narrator and listener cannot be explained by gestures simply appearing together with emotionally salient speech. Instead, the emotional kinematic modulation of gestures seems to play a role in affective alignment between individuals. So, whether storytelling in conversation fulfills its primary purpose—to facilitate a meeting of hearts—depends on a concert of factors.

Inspecting ICE plots, we also found evidence that in creating emotional coupling, gesture expressivity and its kinematic components enter into significant interactions with non-gesture-related situational factors. Such interactions include the interactions of group composition (all-female, all-male, and mixed) and, respectively, group size (dyads v. triads) on the one hand and all seven gesture kinematic parameters on the other. The former interactions suggest that the effects of the gesture kinematics on the achievement of emotional coupling are much greater in all-male groups than all-female or mixed groups. The latter interactions suggest that the influence of gesture dynamics on emotional synchrony are stronger in triadic than dyadic conversational storytellings. We will refrain here from commenting on the association between gender, gender composition, and emotional synchrony. The finding that group size—both in itself and in its interactions with gesture kinematics—impacts resonance suggests that the basic interactional organization of a conversation—whether it is between two or more people—matters fundamentally. Only very few studies have concerned themselves so far with the effects of group size. For example, Holler et al. (2021) found that response times in triads were shorter than dyads due to, the authors argue, competition. This study points to the possibility that group size affects interactional coupling dynamics far beyond just response timing.

When considering multimodal emotional expresssion and physiological synchrony, one important outcome of the Random Forest analysis is the inclusion of both gesture expressivity and speech sentiment as contributing to emotional arousal. Specifically, we show that while the intensity of sentiment conveyed in speech is associated with emotional arousal in the listener, the expressivity of co-occurring gestures also plays a role. This finding highlights the notion of *multimodal expressivity*, and is in line with previous findings that gestures can enhance the emotions conveyed by speech (Asalıoğlu and Göksun, 2023; Guilbert et al., 2021; Levy and Kelly, 2020). Finally, it is important to note that the present quantification of gesture expressivity is derived from a theoretically-motivated mix of kinematic and multimodal-embedding features. It may be useful to explore which other features contribute to an even more meaningful gesture expressivity index. For example, features worth examining in this resepect include fluidity and rhythmicity of movement (Hartmann et al., 2005; Pelachaud, 2009; Pouw et al., 2020; Trujillo et al., 2019).

## 5 Concluding remarks

Storytelling in conversation is an important “body-based way for instantiating a socioemotional connection with another” (Marsh et al., 2009, p. 334). This study has demonstrated that gestures play a key role in establishing that connection: story narrators use gestures skillfully varying their kinematic properties and expressive potentials. The effect of that kinematic virtuosity is emotional resonance: a momentary coupling of emotional affectedness in participants to storytelling—given the deep connections of emotions to life tasks, this coupling represents a powerful way of closing the inherent gap between bodies, brains, and hearts.

Specifically, this study produced three novel findings. First, the kinematic expressivity of gestures increases as the storytelling progresses toward the climax, following the general emotional build-up conveyed through speech and following the basic climacto-telic structure of storytelling. Second, increased gesture expressivity during storytelling increases the probability that participants to the storytelling experience specific EDA responses, that is, responses that reveal gesture-related emotional arousal. Third, gesture expressivity, in all its kinematic diversity, is one important factor contributing to the achievement of emotional resonance between storytelling participants; the most important factor, among a number of non-gesture-related linguistic and situational factors engendering emotional coupling, however, is the gender composition of the storytelling group. We also observed that all gesture kinematics substantially interact with group composition, and also group size, in engendering emotional resonance.

A limitation to this study is that despite the already large number of factors considered it may still not factor in all sources potentially influencing emotional resonance. Factors that future studies would necessarily have to take into account include the participants’ interpersonal dynamics (whether they are strangers, friends, or romantic partners), paralinguistic prosody, which enables speakers to “achieve an infinite variety of emotional, attitudinal, and stylistic effects” (Wennerstrom, 2001, p. 200; cf. also Goodwin et al., 2012; for a study on paralinguistic synchrony see Paz et al., 2021) and also gaze: Kendon (1967), for example, observes hightened emotional arousal in phases of mutual gaze (cf. also Hietanen, 2018). Another factor to examine relates to the “Big Five” personality traits: for example, extroversion (v. introversion) has been shown to correlate with more sizable gestures (e.g., Mehl et al., 2007). Another, probably even more elusive, factor influencing emotional resonance is the extent to which people are *susceptible* to emotional resonance in the first place. The range of factors that may cause individual differences in susceptibility to emotional contagion include for example genetics, early experience, and personality characteristics (Doherty, 1997, p. 133).

Methodologically, this study opens up new avenues of multimodal corpus linguistic research by examining gesture at micro-analytic kinematic levels and using advanced machine-learning methods to deal with the inherent collinearity of multimodal variables. More good is expected to come from this fruitful combination of qualitative and quantitative research.

## Data Availability

The raw data supporting the conclusions of this article will be made available by the authors, without undue reservation.

## Footnotes

^1^EDA responsiveness varies substantially between subjects. This variability is mostly due to physiological conditions (such as the thickness of the cornea); rarer factors impacting on responsiveness include psychopathological conditions (Lykken and Venables, 1971; Dawson et al., 2000).

^2^One story (“Pay more”), which is the shortest in the sample with 10.9 s, consists of exactly *one* a-then-b relation only: *[£I lit]erally had a call today with the health insurance£ £where they were like saying ~ oh we need to update your document (.) you’ll [have to pay] more ~ and I was like ~ (*↑*I do not wanna pay more*↑*)£ ~ [((v: laughs))] ((v: laughs))*, where the two examples of direct quotes, indicated by “~,” represent the two narrative events.

^3^For illustration, cf. the very engaged response tokens *£°aw°£* (line 43) and *holy sh:i::t* (line 48) surrounding the climax in excerpt (1).

^4^The notion of storytelling progression is tied to structural models of storytelling such as Labov’s (1972) model and models underlying conversation-analytic analyses of storytelling. Their common denominator is the assumption that storytelling is a structured activity: “a story is not, in principle, a block of talk” (Jefferson, 1978, p. 245) but falls into “larger structures of talk” (Goodwin, 1984, p. 241) These “larger structures” are the story’s “sections” (Labov, 1972), “segments” (Jefferson, 1978) or “components” (Goodwin, 1984) including Preface, Background, and Climax as well as Post-completion sequences.
